# Integrating screening and management of mental disorders, including substance use disorders into other non-communicable disease care: insights from theory-informed implementation strategies creation for implementation model M0 in Faridabad, India as part of ICMR-MINDS project

**DOI:** 10.3389/frhs.2026.1764829

**Published:** 2026-04-23

**Authors:** Yatan Pal Singh Balhara, Parag Bhardwaj, Siddharth Sarkar, Hitesh Verma, Kuldeep Singh, Gerish Atri, Om Pal Singh Saini, Hanspreet Kang, Pulkit Verma, Ashoo Grover, Neha Dahiya

**Affiliations:** 1 National Drug Dependence Treatment Centre (NDDTC), All India Institute of Medical Sciences (AIIMS), New Delhi, India; 2 Mental Health Services, DGHS Office Panchkula, Panchkula, India; 3 Director General Health Services (DGHS), Haryana, India; 4 ASMO NCD (SPO NP-NCD & NPPC, SNO NPHCE), State NCD Division, NHM Panchkula, Panchkula, India; 5 NCD (NP-NCD, NPHCE & NPPC), State NCD Division, NHM Panchkula, Panchkula, India; 6 WHO NCD Consultant, Panchkula, India; 7 Data Centre, Indian Council of Medical Research Headquarters, New Delhi, India; 8 Delivery Division, ICMR Hqrs, New Delhi, India

**Keywords:** health planning, health services accessibility, implementation science, integrated health care systems, mental disorders, non-communicable diseases, primary health care, substance use disorders

## Abstract

**Background:**

Mental disorders, including substance use disorders (MSUD) frequently co-occur with other Non-Communicable Diseases (NCDs). This leads to increased morbidity, premature mortality, and reduced quality of life. In India, services for MSUD are usually delivered separately from NCD care. This study aimed to develop a theory-informed and context-specific set of implementation strategies as part of Model M0 for integration of screening and management of MSUD into existing NCD care in public health facilities in Faridabad district of Haryana. This work addresses a major service gap in the public health system and provides a structured, practical approach for integration.

**Methods:**

Implementation Mapping, updated Consolidated Framework for Implementation Research, Expert Recommendations for Implementing Change (ERIC) taxonomy, Theoretical Domains Framework and Capability, Opportunity, Motivation – Behavior model were used to design and tailor implementation strategies. Mixed-methods formative assessment was carried out. The stakeholders (actors) included the health system leaders (policy makers and state and district health authorities), facility-level healthcare professionals, and patients/service users and caregivers. The barriers, facilitators, and determinants were identified. Co-creation meetings were held with stakeholders. A set of ERIC strategies were operationalized through contextually appropriate actions and materials.

**Results:**

A comprehensive, theory-informed implementation model (Model M0) integrating 51 ERIC strategies across domains such as capacity building, clinical workflow optimization, stakeholder engagement, and data systems strengthening was created. Multiple co-creation meetings conducted with various stakeholders at the level of state, district, and health facility benefited from and incorporated the perspectives and inputs from them. Strategies were mapped to specific change objectives and stakeholders, including patients/service users and caregivers, health care professionals, and health system leaders (policy makers and state and district health authorities). Specific actions and target actors (stakeholders) for each of the strategies were identified. The model M0 included the set of implementation strategies; the interventions (innovations), implementation materials (practical tools and protocols) and indicators to assess process, implementation, patient/clinical, and service outcomes.

**Conclusions:**

This study demonstrates the feasibility of applying a structured implementation science approach to design context-sensitive strategies for integrating services for MSUD into existing NCD care in public health facilities in Faridabad district of Haryana. The implementation Model M0 offers a clear roadmap for how integration can be carried out in routine practice. The recommended way forward is to pilot, review, and refine this model. This will be followed by scale-up within the district and evaluation. The approach may also be useful for other low- and middle-income countries aiming to strengthen integrated care for MSUD within NCD programs.

**Clinical Trial Registration:**

https://ctri.nic.in/Clinicaltrials/pmaindet2.php?EncHid=MTEzMTg4&Enc=&userName=, identifier CTRI/2024/08/072748.

## 1 Background

Mental disorders, including substance use disorders (MSUD), co-occurring with other non-communicable diseases (NCDs) contribute to the burden due to NCDs. This includes indicators such as prevalence, disability-adjusted life years (DALYs), and premature mortality. MSUDs have been reported to increase NCD-related premature deaths by 40%–60% (1). In addition, NCDs have increased the health system burden by creating long-term demand for continuous care, follow-up, and resource use.

NCDs are the leading cause of mortality and morbidity in India. Hence, it is important to address the MSUD among people living with other NCDs (2, 3). This is critical to achieve the Sustainable Development Goal (SDG) target of reducing premature mortality from NCDs by one-third by 2030 through prevention and treatment (4). It has also been proposed that the quality of life of patients with NCDs will improve when MSUD and other NCDs are managed consistently (5). However, the treatment gap for any MSUD in India was reported to be as high as 83% (6, 7).

Currently, services for MSUDs and other NCDs in India are delivered through two programs. While the need to have an integrated approach in the context of MSUD and other NCDs has been expressed earlier, the real world implementation of the same remains limited (8). This leads to missed opportunities for early identification, and comprehensive and continued care for MSUDs (9, 10). The National Programme for Prevention and Control of Non-Communicable Diseases (NP- NCD) is one of the flagship programs in India under the National Health Mission (NHM). Around 770 District NCD Clinics have been established under the program. Currently, MSUD care is not integrated in this. At the national level services for MSUD are offered primarily through the National Mental Health Program (11).

To address this critical gap, the Indian Council of Medical Research (ICMR) initiated the Implementation Research Study on Integration of Screening and Management of Mental and Substance Use Disorders with Other Non-Communicable Diseases [ICMR-MINDS, (12)]. ICMR- MINDS is one of the National Health Research Priority projects of ICMR [Indian Council of Medical Research, (12)]. Focusing on implementation research is essential to bridge the gap between evidence-based MSUD interventions and their successful delivery in real-world settings.

Implementation science provides a set of theories, models, and frameworks that help understand and address real-world challenges to delivering evidence-based care. Frameworks such as the Consolidated Framework for Implementation Research (CFIR) and Implementation Mapping (IMap) support systematic identification of contextual factors, stakeholder needs, and implementation strategies. These approaches help ensure that interventions are not only based in theory but also delivered in an acceptable, practical, and scalable way within routine health systems.

The objective of this article is to document the process of development of a set of implementation strategies that will be included in the initial implementation Model (Model 0 or M0). The model aims at integration of screening and management of MSUD within existing NCD care in Faridabad District of Haryana state. The Model M0 will essentially be the first version (the prototype implementation model) before actual field implementation begins. It will serve as the blueprint for how integration will work in practice. This model will contain the set of implementation strategies; the innovations (intervention), implementation materials (practical tools and protocols) and indicators to assess process, implementation, patient/clinical, and service outcomes for integrated care for MSUD in the existing care for NCD in Faridabad district of Haryana. The purpose of M0 will be to translate theory into practice. It will also provide a structured foundation for subsequent piloting and refinement.

This article is expected to generate insights for policy-makers, program managers (administrators), health care professionals, researchers, academics aiming to bridge the mental-physical health divide globally, and specifically in low- and middle-income country (LMIC) settings.

## 2 Methods

### 2.1 Overview of the parent study

The Implementation Research Study on Integration of Screening and Management of Mental and Substance Use Disorders with Other Non-Communicable Diseases (ICMR-MINDS) study is an implementation research project. ICMR-MINDS has been recognized as one of the National Health Research Priority projects by the Indian Council of Medical Research [ICMR, (13)]. It adopts a mixed-methods, quasi-experimental, single-arm, interrupted time series design with three sequential phases. These phases are the Formative Research, Model Optimization, and Full Implementation and Evaluation (12). The current article is based on the work done as part of the Formative phase of the project.

### 2.2 Study setting

The study was conducted in Faridabad district, located in the Indian state of Haryana (14). The district has a population of more than 2.4 million. The selection of Faridabad district was made in consultation with state health authorities. Faridabad encompasses both rural and urban regions. Its proximity to the host institution of the principal investigators was a key factor. This was critical given the study's requirement for extensive field level engagement. Previous health system research has highlighted Faridabad as one of the weaker-performing districts in Haryana across the domains of health financing, human resources, facility readiness, Health Management Information System (HMIS), service delivery, and governance (15). This presented an opportunity to develop and test the model in a setting with known implementation challenges. Successful demonstration of the model in such a context could support its broader adoption across the state and other settings (14).

#### 2.2.1 Identification of public health facilities

Approval to conduct the study was obtained from the National Health Mission (NHM), Government of Haryana. A comprehensive list of public sector health facilities registered with the district's Chief Medical Officer (Civil Surgeon) was compiled by the study team. It was verified against the administrative records before inclusion. This list included facility names, addresses, and levels of care. All public health facilities in Faridabad district (totaling 116 facilities) were included in the study. These comprised Aayushman Arogya Mandir- Sub Centres (AAM-SC), Aayushman Arogya Mandir- Primary Health Centres (AAM-PHC), Community Health Centres (CHC), Sub-Divisional (Sub-District) Hospital, and the District Hospital.

### 2.3 Inclusion and exclusion criteria

The healthcare professionals aged 18 years and above, of all cadres across healthcare facilities in Faridabad district of Harayan state involved with NCD and Mental Health service delivery at various levels of healthcare delivery, interested in participating in the study and willing to provide consent for the same, were eligible for inclusion in the study. Those refusing to consent or not interested in participating in the study were excluded.

### 2.4 Co-creation paradigm

The implementation strategies for the study were developed using a co-creation approach. This approach in implementation science fosters partnerships between researchers and other actors (stakeholders). It facilitates the involvement of end-users in research design, enhances their understanding and capacity, and encourages the uptake of findings (16, 17). The study included key implementation science principles facilitated by a co-creation approach. This was guided by IMap. This process involved systematic, theory-informed, and participatory development of implementation strategies. Frameworks such as the Consolidated Framework for Implementation Research (CFIR), Theoretical Domains Framework (TDF), COM-B (Capability, Opportunity, Motivation-Behaviour) model, Diffusion of Innovations Theory Self-Determination Theory (SDT) and Expert Recommendations for Implementing Change (ERIC) were used to identify determinants, link them to behavior-change methods, and collaboratively design context-specific solutions with stakeholders. These have been used in the past for such purposes (18–26). This ensured that all strategies were both evidence-based and grounded in the realities.

### 2.5 Implementation mapping (IMap)

A sequential approach guided by the Implementation Mapping (IMap) methodology was utilised (27). This approach was used to develop, choose, and create a set of implementation strategies specifically for implementing the proposed innovations (interventions) for the project (28). IMap was selected as the overarching framework because it provides a structured, theory-informed process for developing and tailoring implementation strategies. IMap integrates findings from needs assessment into a stepwise logic model linking outcomes, performance objectives, determinants, change methods, and strategies.

**Figure 1:**
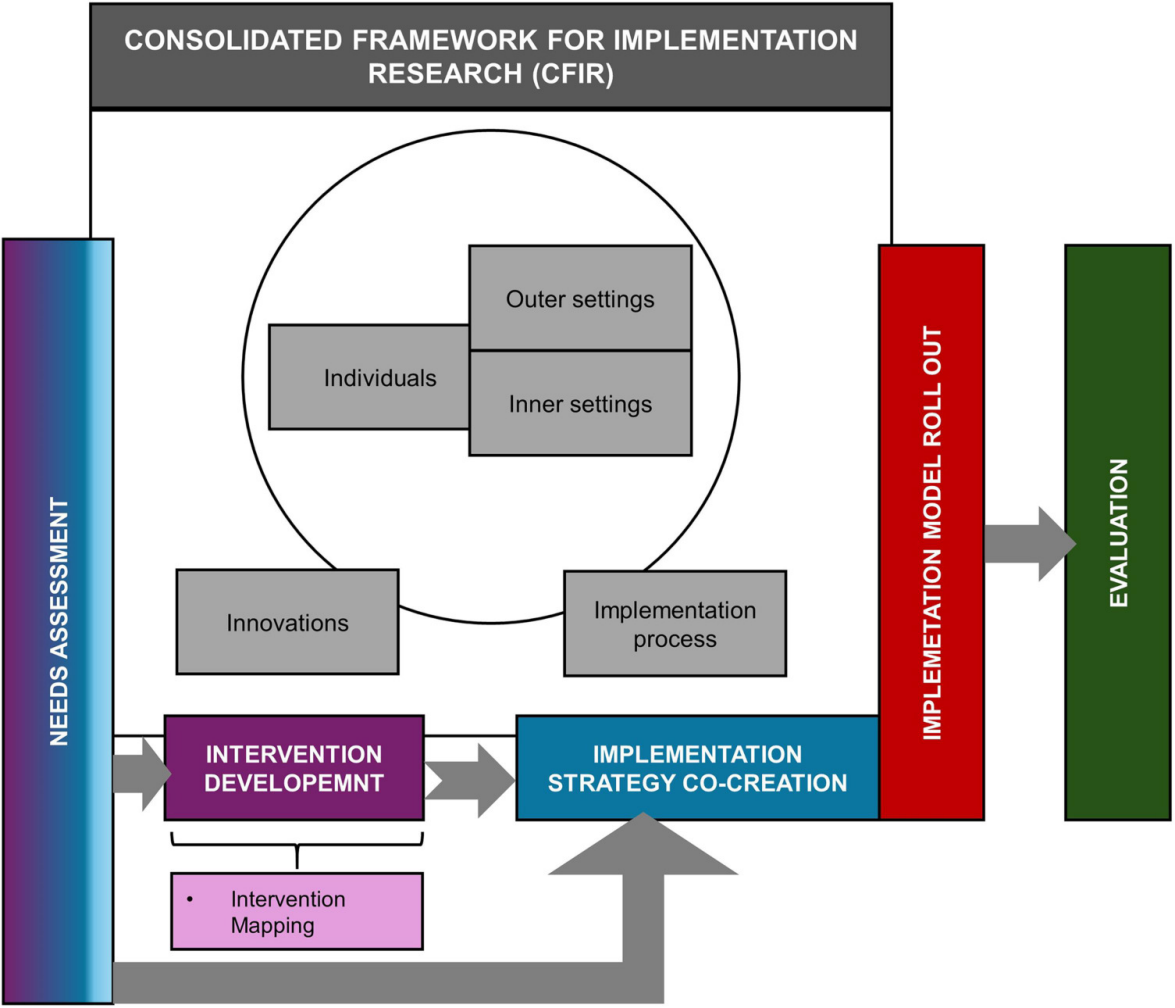
CFIR framework based approach guiding the various aspects of the project in the faridabad district of haryana as part of ICMR-MINDS project.

The choice of IMap was guided by various reasons. First, IMap can be used alongside frameworks such as CFIR, rather than being constrained by them (18, 19). Also, it leverages on the findings from the needs assessment exercise that forms one of the key elements of the CFIR-based determinant framework (Figure 1).

IMap involves five specific tasks including conducting a needs assessment and identifying innovation adopters and implementers; stating adoption and implementation outcomes and performance objectives identifying determinants, and create matrices of change objectives; choosing theoretical methods and select or design implementation strategies; producing implementation protocols and materials; and evaluating implementation outcomes. For the current study we employed the first four steps. We adapted the approach followed by Fakha et al. for this (29). They utilised multiple taxonomies and overviews of change methods as well as relevant evidence on their effectiveness to select the implementation strategies targeting each of the relevant factors for their study. The Tasks of IMap, objectives and specific approaches have been summarised in Table 1.

**Table 1:** Tasks of implementation mapping, objectives and specific approach for the current study.

Task Number	Name of the tasks	Objectives	Approach
1	Conducting a needs assessment and identifying innovation adopters and implementers	Assess the implementation context Identify barriers and facilitators to implementation Engage key stakeholders early Clarify the implementation problem or gap Identify intended innovation adopters Identify implementers and end users Collect and analyze data to inform planning Map stakeholder roles and relationships	Literature review, baseline assessment using mixed methods approach (qualitative and quantitative designs)
2	Stating adoption and implementation outcomes and performance objectives, identify determinants, and create matrices of change objectives	Specify desired adoption and implementation outcomes Identify actors responsible for each outcome Develop performance objectives for adopters and implementers Identify determinants influencing behavior Link performance objectives to determinants Create matrices of change objectives Ensure change objectives are measurable and actionable Use theoretical and empirical evidence to support selections	Data from literature review, baseline assessment using mixed methods approach (qualitative and quantitative designs) Various actors (stakeholders) as part of co-creation exercise Expert group meetings
3	Choosing theoretical methods and select or design implementation strategies	Select theoretical methods that can influence determinants Ensure theoretical methods match determinants and change objectives Identify parameters for effective use of theoretical methods Translate theoretical methods into practical implementation strategies Select or design implementation strategies for each change objective Tailor strategies to context and stakeholders Ensure strategies address multiple levels of influence Document rationale for strategy selection Link strategies back to matrices of change objectives	Literature on theoretical change methods Literature for evidence on the effectiveness of innovations (interventions) Various stakeholders as part of co-creation exercise Inputs from experts in the fields of implementation science, mental health, addictive disorders, public health
4	Producing implementation protocols and materials	Translate implementation strategies into actionable protocols Design and develop implementation materials Ensure alignment with theoretical methods and determinants Tailor materials to target audiences Pilot test protocols and materials Revise based on stakeholder feedback Document implementation processes clearly	Various stakeholders as part of co-creation exercise Experts in the fields of implementation science, mental health, addictive disorders, public health

#### 2.5.1 Use of implementation mapping in co-creation meetings

IMap was used as a practical guide to structure and conduct co-creation meetings with stakeholders. Each meeting followed a predefined agenda aligned with specific IMap steps. Structured worksheets and matrices were used to ensure systematic discussion and documentation.

For the IMap Task 1, stakeholders reviewed the summarized findings from the quantitative and qualitative needs assessment using guided discussion prompts. These discussion prompts focused on identifying key barriers and facilitators to integrating MSUD care into existing NCD services at different facility levels.

For IMap Task 2, matrices of change objectives were used. Stakeholders worked with structured worksheets that linked performance objectives (what health care professionals or the system need to do) with determinants identified through CFIR, TDF, and COM-B frameworks. Facilitators guided stakeholders to identify what needed to change in knowledge, skills, motivation, resources, or workflows to achieve each performance objective.

For IMap Task 3 and Task 4, stakeholders used prompts to select and refine theory-informed implementation methods and translate them into feasible strategies. Matrices were used to map determinants to potential strategies. In addition, discussions focused on feasibility, acceptability, and alignment with existing health system structures. The relevant implementation materials were also created using a similar approach.

#### 2.5.1 Stakeholder mapping and engagement

**Figure 2:**
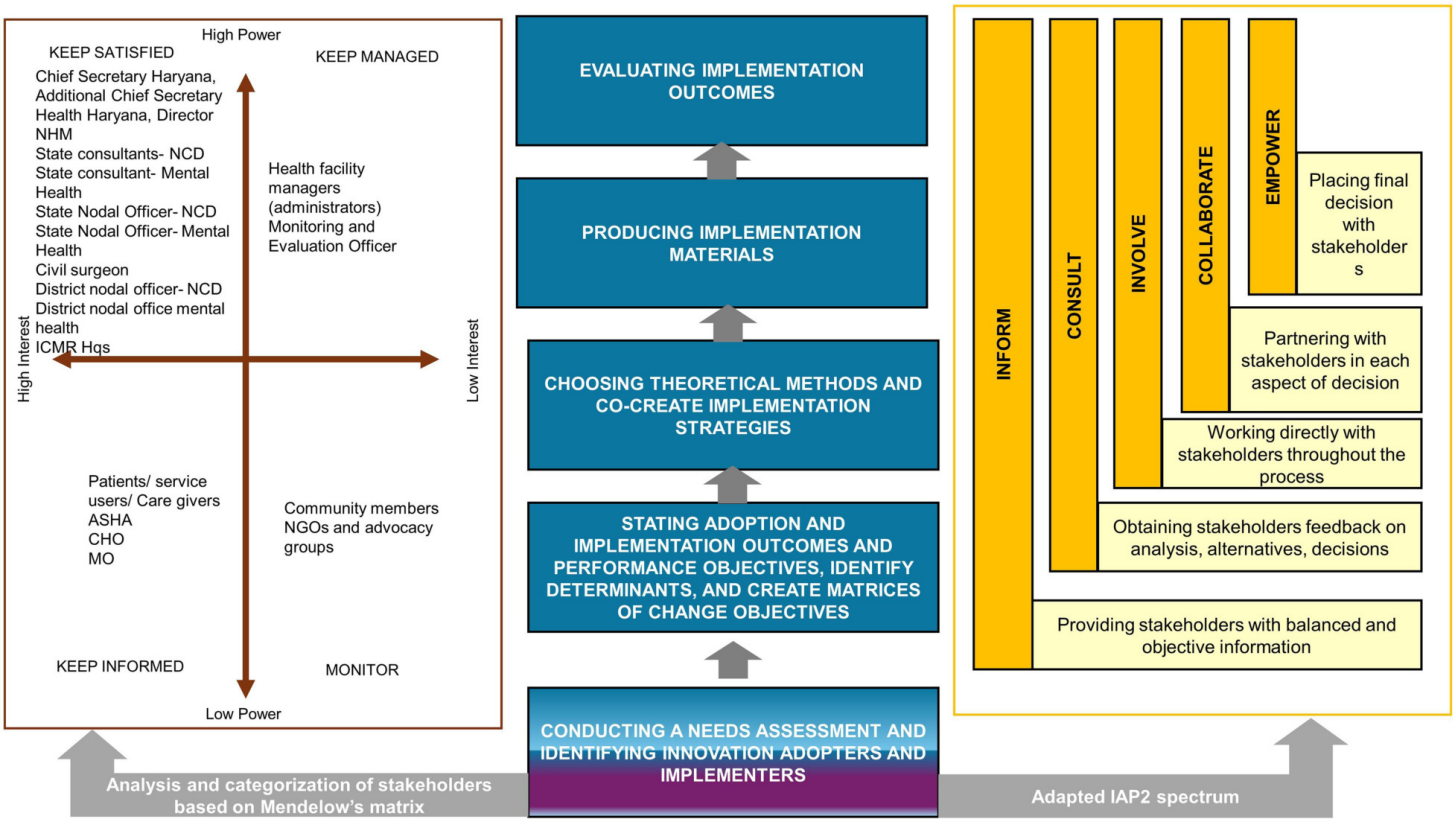
Tasks carried out as part of IMap, Mendelow's matrix (stakeholder mapping matrix) and international association for public participation (IAP2) recommendation used during IMap.

Mendelow's Matrix (Stakeholder Mapping Matrix) was used to analyze and categorize actors (stakeholders) based on their level of power and interest in the health system (30). Mendelow's Matrix categorises the stakeholders into four groups including keep satisfied (stakeholders with high power, high interest); keep managed (stakeholders with high power, low interest); keep informed (stakeholders with low power, high interest); and monitor (stakeholders with low power, low interest). The recommendations of the International Association for Public Participation (IAP2) to enhance stakeholder engagement were also utilised (31). IAP2 provides a set of core recommendations for effective stakeholder engagement through its Public Participation Spectrum. This framework outlines five levels of engagement including inform, consult, involve, collaborate, and empower. Each of these levels represents increasing levels of public influence in decision-making. These recommendations helped ensure that participation was meaningful, inclusive, and aligned with the goals of both decision-makers and the affected persons (Figure 2).

Multiple co-creation meetings were conducted with various stakeholders at the level of state, district, and various public health facilities. Co-creation meetings were facilitated by members of the project team. Discussions were guided by predefined prompts and worksheets aligned with specific IMap tasks. To build consensus, facilitators encouraged open group discussion to generate options. This was followed by prioritising where participants prioritised barriers, facilitators, determinants, and strategies based on feasibility and perceived impact. The emphasis was to build a consensus among the stakeholders. In cases of disagreement, facilitators used iterative feedback and revisited the options in subsequent meetings after incorporating stakeholder suggestions. Consensus was considered achieved when there was broad agreement and no major objections among participants.

The co-creation meetings enabled the development of implementation strategies within the local context. The sessions led to translation of barriers and facilitators identified through the Consolidated Framework for Implementation Research (CFIR) into practical, context-specific actions. These actions were aligned with ERIC strategies. The participants adapted theoretical methods to local workflows, resources, and norms through iterative discussions and consensus-building. This ensured that the resulting implementation strategies were feasible, acceptable, and sustainable within the public health system in Faridabad district.

#### 2.5.2 Task carried out as part of IMap

This section outlines the key tasks undertaken as part of IMap and explains how these guided the development of implementation strategies.

##### 2.5.2.1 Task 1 conducting a needs assessment and identifying innovation adopters and implementers

For the first Task a needs assessment was carried out using a combination of desk review of published research and original data collection. The desk review aimed to gather information on policy and strategic context, health system infrastructure, service delivery financing, resource allocation, workforce capacity, training program, available interventions, community and sociocultural context, and evidence gaps. It also aimed to identify the potential barriers and facilitators to the implementation of the project.

The original data were collected using a mixed- methods approach. It was guided by the updated Consolidated Framework for Implementation Research [CFIR, (19, 32)]. During instrument development, CFIR domains were used to structure the interview and survey guides. This ensured comprehensive exploration of determinants influencing integration of MSUD into existing NCD care. The questions and indicators were mapped to a relevant CFIR construct. During data analysis, CFIR guided the thematic coding framework. This allowed qualitative data to be systematically categorized by determinant domains. Qualitative and quantitative findings were triangulated to identify multilevel barriers and facilitators. These were then mapped to CFIR domains [innovations (interventions), outer setting, inner setting, characteristics of individuals, and implementation process]. This ensured that all variables were theoretically grounded and directly informed the selection of implementation strategies in subsequent IMap tasks. Together, these methods aimed to provide a comprehensive picture of system-level and stakeholder-specific needs for integration of services for MSUDs into the existing NCD care. Additionally, it helped to determine the barriers and facilitators of implementation.

###### 2.5.2.1.1 Quantitative data

Quantitative data were collected using a structured questionnaire across 116 public health facilities. The information was gathered on service availability (for MSUD), human resources, infrastructure (e.g., availability of consultation rooms, privacy for counseling, and dedicated space for MSUD services), medication supply, data systems, referral mechanisms (for patients across public health facilities at different levels such as primary, secondary and tertiary). The questionnaire was developed by the ICMR-MINDS group (12). The questionnaires were administered in-person to facility heads or senior staff. Quality assurance was ensured by the use of a structured proforma for data collection, enumerator training (training field workers thoroughly on study objectives and ethics, standardized data collection protocols, explaining the questionnaire neutrally, managing respondent fatigue and maintain data privacy), conducting mock interviews, random spot checks and accompanied interviews, review of forms for missing data, outliers, and inconsistent responses, and in-built features of digital database.

###### 2.5.2.1.2 Qualitative data

Qualitative data were collected through in-depth interviews (IDI) and focus group discussions (FGD). IDIs and FGDs were used complementarily. Using both methods strengthened triangulation and improved identification of multilevel implementation determinants.

IDIs enabled in-depth exploration of individual experiences, sensitive issues, and personal beliefs. In-depth interviews were conducted with four participants each from patients/service users, Medical Officers (MOs), Community Health Officers (CHOs), and Accredited Social Health Activists (ASHA) workers. These represented the various cadres of health care professionals and the service users at the public health facilities in the district.

FGDs captured shared norms, group dynamics, and collective practices. Three FGD were conducted. This included one FGD with CHOs (included in IDIs as well) and two with ASHA workers (not included in IDIs). The FGDs with CHOs included 12 participants and with ASHAs included 14 participants. Due to logistical challenges, focus group discussions with patients/service users and MOs could not be conducted. We aimed to achieve data saturation. Sample sizes were justified based on achieving thematic saturation (33). This was ascertained when no new codes emerged after two additional interviews. Interviews followed a semi-structured guide informed by CFIR constructs. They were audio-recorded with consent, transcribed and translated. Interviews followed a structured protocol for coding. Additional insights were gathered from interviews with key district and state-level health officials and program staff. These interviews were planned *a priori* and carried out in the initial phase.

###### 2.5.2.1.3 Data analysis

Descriptive analysis of the quantitative data (frequency and percentage) was performed using Microsoft Excel. Qualitative data were coded in Atlas.ti v9 using a structured codebook derived from CFIR constructs. Coding discrepancies were resolved through discussion involving a senior researcher. An iterative process was followed for Tasks 2–4 and amendments were made to reach the implementation strategies that will be integrated in Model M0.

##### 2.5.2.2 Task 2 stating adoption and implementation outcomes and performance objectives, identify determinants, and create matrices of change objectives

Task 2 of IMap led to specification of the desired implementation and adoption outcomes specific to adopters and implementers identified in Task 1. It also identified key determinants that influenced those outcomes, and created matrices linking performance objectives (POs) to determinants. Additionally, it created change objectives (COs) by crossing implementer-specific POs with relevant determinants. This ensured strategies developed in later tasks were explicitly theory-based and targeted identified barriers and facilitators to support the integration of MSUD care into the existing NCD care. The choice of determinants was guided by the CFIR, Theoretical Domains Framework (TDF), COM-B Model (Capability, Opportunity, Motivation—Behavior). It was supplemented by Diffusion of Innovations Theory, Organizational Development Theory and Self-Determination Theory (SDT). This provided a comprehensive, evidence-based foundation for implementation strategy development.

##### 2.5.2.3 Task 3 choosing theoretical methods and co-create implementation strategies

In Task 3 theory-based methods and practical strategies to address the COs defined in Task 2 were identified. This process was guided by the Theoretical Domains Framework (TDF) and COM-B model to choose change methods known to influence target determinants and COs (19–21). This was supplemented by use of Diffusion of Innovations Theory, Organizational Development Theory and Self-Determination Theory (SDT) to enhance contextual understanding of implementation dynamics (24, 25). The implementation strategies were selected from the ERIC strategy compilation [taxonomy, (26)]. First, the theoretical and behavioral methods were identified. Then the research team and stakeholders used these as anchors to select appropriate ERIC strategies. Each potential strategy was critically reviewed for contextual relevance, feasibility, and theoretical alignment with identified determinants. During the co-creation meetings, stakeholders deliberated on the strategies based on fit with local barriers and facilitators identified through the CFIR- guided needs assessment. Feasibility within existing human resources, workflows, and health system structures; and acceptability to implementers and health system leaders (policy makers and state and district health authorities) was also prioritised. Only strategies that met consensus across these criteria were retained. This decision process ensured that selected ERIC strategies were tailored through local validation to align with behavioral methods and contextual realities.

##### 2.5.2.4 Task 4 producing implementation materials and protocols

Task 4 focused on development of practical tools, materials, and protocols guided by the set of implementation strategies created in Task 3 to support the integration of MSUD services into the existing NCD care. These contents were developed around the set of interventions (innovations) that were created by the ICMR- MINDS group (12). These included content aimed at patient education and engagement to raise awareness and promote shared decision-making; digital platform and smartphone app; tools for integrated screening and assessment for mental health conditions; training of HCPs; workflows and pathways that integrate mental health interventions within other NCD care. The practical tools, materials, and protocols were pilot tested in the Tigaon block by the research team in collaboration with the health care professionals. A multidisciplinary panel of technical experts was engaged to review the draft implementation materials for accuracy, usability, and alignment with existing health system protocols. The experts represented domains of psychiatry, addiction psychiatry, digital health and informatics: public health and health systems, and NCD.

The process of creation of the Model M0 based on the implementation strategies was also guided by the findings from the Intervention Mapping (IM). IM is a protocol that guides the design of multi-level health interventions (28). It facilitated developing strategies to enhance the adoption, implementation, and maintenance of interventions. IM was used for the substantiation of the intervention in the local context in Faridabad district. The innovations (interventions) created by the ICMR-MINDS group as part of the project and their substantiation in the Faridabad district have been explained in detail elsewhere (article under review).

The Standards for Quality Improvement Reporting Excellence (SQUIRE) 2.0 guidelines were followed for reporting new knowledge about how to improve healthcare [attached as Supplementary File, (34)]. The study was approved by the institute ethics committee. Written, informed consent was taken from the participants before inclusion in the study.

## 3 Results

### 3.1 Stakeholder mapping and engagement and use of co-creation paradigm

The Chief Secretary of Haryana, Additional Chief Secretary Health Haryana, Director NHM, State Consultants for NCD, State Consultant for Mental Health, State Nodal Officer for NCD, Assistant State Medical Officer for NCD, State Nodal Officer for Mental health, District Civil Surgeon Faridabad district, Deputy District Civil Surgeon Faridabad district (nodal officer for Mental Health), District nodal officer- NCD, ICMR Hqs were identified as stakeholders with high power, high interest. The MO, CHO, ASHA workers and patients/service users and caregivers were identified as stakeholders with low power, high interest. The health facility administrators and the monitoring and evaluation officer were identified as stakeholders with high power, low interest. Community members, civil society organizations and advocacy groups were identified as stakeholders with low power, low interest. The recommendations of the International Association for Public Participation (IAP2) were adapted to enhance stakeholder engagement (Figure 2).

Multiple co-creation meetings were conducted with various stakeholders at the level of state, district, and health facilities. A summary of some of these co-creation meetings is presented in Supplementary Table S1. The details of the co- creation meeting for various Tasks has been presented in the sections on respective Tasks.

### 3.2 Findings from needs assessment

#### 3.2.1 Barriers and facilitators

The availability of MSUD services varied across public health facilities. Screening for mental disorders was reported in 29% of AAM-SC and 75% of CHCs. Screening for substance use disorders ranged from 22% of AAM-SC to 58% of CHCs. Counselling services were available in fewer than 55% of health facilities, and essential medicines for MSUDs were limited.

Between 25%–39% of health facilities reported having HCP trained in MSUD management. Gaps were also observed in system readiness. Most facilities lacked MSUD-specific record-keeping systems, structured referral and back-referral mechanisms, and consistent availability of essential medicines.

Qualitative findings explained the quantitative gaps and highlighted lived experiences of healthcare professionals, patients, and caregivers. The healthcare professionals reported limited training, limited access to tools, and unclear care pathways. As a result, many preferred referring patients rather than managing them locally. Healthcare professionals expressed that regular and practical training would improve their ability to deliver MSUD care confidently.

Strong stigma related to mental illness and substance use emerged as a major barrier at the community level. Patients and caregivers described fear of social labelling, isolation, and discrimination, which reduced disclosure and delayed care-seeking. Financial difficulties, travel distance, and cost of medicines further limited access to services. Families sometimes discouraged engagement with health workers due to fear of being identified as having a mental health problem.

Within health facilities, health care professionals highlighted system-level barriers such as competing clinical priorities, high workload, lack of private space, weak referral linkages, and absence of standard workflows. Despite these challenges, several facilitators were identified. Healthcare professionals across cadres expressed strong interest in receiving training and playing a greater role in MSUD care. District- and state-level stakeholders supported service integration and pointed to alignment with national programs such as NP-NCD and initiatives such as TeleMANAS. Stakeholders emphasized the need for brief tools, clear role definitions, simple digital systems, and community engagement to improve acceptance and uptake of integrated MSUD services.

The focus areas that emerged for implementation strategy development included stakeholder knowledge and skills; clinical infrastructure; service delivery gaps; human resources; referral and continuum of care; data collection and Health Information Technology (HIT); community engagement; and medication availability. Various barriers and facilitators to the creation and execution of implementation strategies were also identified for each area. These have been summarised in Table 2.

**Table 2:** Barriers and facilitators for creation and execution of implementation strategies for integration of MSUD care in the existing NCD care in faridabad district of haryana.

Focus areas	Barriers Identified	Facilitators Identified
Stakeholder knowledge and skills	Low awareness of MSUDs among HCPs due to limited or no training	Some facilities reported prior training, interest and perceived benefit of training
Clinical infrastructure	Lack of separate space for screening and assessment, few facilities had guidelines or tools	DH and select AAM- PHCs had some infrastructure, indicating partial readiness
Service delivery gaps	MSUD care not part of NCD workflow, no dedicated time or tools for screening	Existing NCD service platforms were functional and could be leveraged
Human resources	No psychiatrist appointed in the district	CHOs and ASHAs were already engaged with the existing NCD care
Referral and continuum of care	Limited formal referral pathways, lack of feedback loops from higher centres	Informal telephonic referrals existed at some PHCs and CHCs
Data collection and Health Information Technology (HIT)	No separate registers or digital records for MSUDs	Digital literacy and smartphone use among staff was high
Community engagement	High stigma and limited awareness, limited structured community linkages	ASHAs and ANMs had community trust and could be engaged in awareness activities
Medication availability	Medicines for treatment of MSUD not available in all facilities	Availability of some of the medicines at the district hospital and some other facilities

MSUD- Mental disorders, including substance use disorders; DH- District Hospital; CHO- Community Health Officers (CHO); ASHA- Accredited Social Health Activists; DGHS- Director General of Health Services; NCD- Non Communicable Diseases; MH- Mental Health; AAM PHC- Aayushman Aarogya Mandir Primary Health Centre; CHC- Community Health Centre; ICMR- Indian Council of Medical Research; DG- Director General; HCP- Health Care professionals.

#### 3.2.2 Specific implementation factors, innovation adopters and implementers

The specific implementation factors were identified and categorised as per the updated CFIR. Each barrier, facilitator, and contextual factor emerging from the qualitative and quantitative data was categorized under relevant CFIR domains [innovations (interventions), inner setting, outer setting, individuals, and process]. This mapping provided a structured understanding of how various factors such as organizational culture, leadership engagement, resource availability, provider knowledge, and workflow processes affected readiness for implementation. The specific implementation factors and their corresponding CFIR domains have been listed in Supplementary Table S2.

Co-creation meetings served to validate formative findings and refine the understanding of local barriers and facilitators. Preliminary qualitative and quantitative results were presented to stakeholders to verify accuracy and completeness. Participants identified key adopters and implementers, clarified workflow bottlenecks, and prioritized determinants most relevant to integration of MSUD services into existing NCD care. These discussions ensured that subsequent mapping of determinants reflected ground realities.

### 3.3 Task 2 stating adoption and implementation outcomes and performance objectives, identify determinants, and create matrices of change objectives

Each of the implementation factors determined in Task 1 was linked to its equivalent determinants (Supplementary Table S2).

#### 3.3.1 Adoption and implementation outcomes and performance objectives

The desired adoption and implementation outcomes were identified. The choice of these outcomes was guided by the study protocol (12). This included the uptake of the implementation model and fidelity of implementation by healthcare professionals. Next, for each outcome, performance objectives (POs) were developed. The POs guided the specific actions that key actors (stakeholders) must carry out to achieve successful adoption and implementation of our model (listed in Task 3).

#### 3.3.2 Identification of determinants

The determinants that influence the POs were identified. The choice of these determinants was guided by findings from the needs assessment (Task 1) and relevant theoretical constructs. Determinants influencing each PO were identified through triangulation of CFIR constructs and complementary theories [Theoretical Domains Framework (TDF); Capability, Opportunity, Motivation–Behavior (COM-B); supplemented by Diffusion of Innovations Theory, Organizational Development Theory and Self-Determination Theory]. Cross-walk (matrix) of outcomes, adopters/implementers, POs, and determinants were prepared. Matrices were constructed for each outcome. A few sample matrices are presented in Supplementary Table S3.

#### 3.3.3 Matrices of change objectives (COs)

The matrices of change objectives (COs) were created by mapping each PO against its relevant determinants to generate specific COs (Supplementary Table S4). These matrices served as the foundation for selecting theoretical methods and implementation strategies in Task 3. This helped to ensure that implementation strategies were precisely targeted and grounded in the theory and were evidence-based.

The co-creation meetings facilitated joint definition of implementation outcomes and POs for each cadre of implementer. Stakeholders collaboratively refined measurable objectives (e.g., routine screening by CHOs, assessment by MOs) and validated the determinants mapped to the CFIR and TDF frameworks. This collaborative refinement increased ownership and alignment with local operational indicators.

### 3.4 Task 3 choosing theoretical methods and co-create implementation strategies

#### 3.4.1 Determinants, implementation factors, theoretical methods, and implementation strategies

The relevant theoretical methods known to influence specific determinants and the implementation strategies have been presented in Supplementary Table S5. Since the determinants (equivalent theoretical construct) overlapped across the change objectives, the findings have been categorised according to the determinants.

The relevant theoretical methods were translated into implementation strategies that were context-specific and actionable. Strategies were selected from the ERIC compilation and customized to align with the practical realities of the public health system in the Faridabad district. We identified a total of 52 ERIC strategies to be part of Model M0. It was decided not to incorporate the strategy of altering incentive/allowance structures as recommended by the ICMR- MINDS group.

As part of co- creation, the stakeholders reviewed the set of ERIC strategies and collectively assessed each option for relevance, feasibility, and acceptability. Participants also contextualized theoretical change methods by specifying practical actions (e.g., adapting training schedules, designing local feedback loops) that could realistically be implemented within existing resources. This transformed the behavioral constructs into concrete, locally relevant strategies. This Task resulted in a theory-informed set of implementation strategies designed to influence individual and systemic behaviors necessary for the implementation model M0.

#### 3.4.2 Adaptation of theoretical methods

Theoretical methods were adapted to fit routine service delivery. For example, the method of simplification and environmental restructuring was applied by integrating MSUD screening tools into existing NCD patient workflows, rather than introducing separate clinics. Screening and assessment was designed to occur during routine NCD visits, using tools appropriate for different cadres (ASHAs, CHOs, and MOs).

Similarly, the theoretical method of guided practice and skill-building was adapted by planning training sessions that focused on screening, assessment, diagnosis and management of MSUD. Decision support was embedded within a digital platform (ICMR-MINDS CDSS) to guide healthcare professionals step-by-step (article under review). This reduced reliance on memory and specialist availability.

Cultural norms, local resources and service delivery realities strongly influenced strategy design. Because patients and families were often reluctant to openly discuss mental health problems, strategies emphasised non-judgemental communication and linking MSUD screening to physical health visits to reduce fear of labelling. Health care professionals' trusted position in the community was leveraged to support awareness and follow-up.

Resource constraints such as limited staff time, medicine shortages, and lack of private space shaped the choice of task-sharing, brief interventions, and stepped-care approaches. For example, ASHAs and CHOs were assigned roles in identification, counselling, brief interventions, referrals and follow-up. MOs focused on assessment and management. Digital tools (ICMR-MINDS CDSS and dashboard) were prioritised to address weak paper-based data systems and limited supervisory capacity.

Service delivery realities such as high patient load and weak referral feedback led to strategies that prioritised clear workflows and simple referral pathways. These adaptations were intended to ensure that implementation strategies were feasible, acceptable, and aligned with routine practice.

#### 3.4.3 Specific actions and corresponding target actors (stakeholders)

The specific actions and corresponding target actors (stakeholders) for each of the strategies were also identified (Supplementary Table S6).

### 3.5 Task 4 producing implementation materials and protocols

The change objectives and matched implementation strategies identified in Task 3 were reviewed. For each strategy, corresponding implementation materials were developed. The content and presentation of the material were developed for each of the five innovations (interventions) that were created by the ICMR-MINDS group. The interventions created as part of the project have been explained in detail elsewhere (article under review). In brief, they included a set of five interventions namely content aimed at patient education and engagement to raise awareness and promote shared decision-making; digital platform and smartphone app; tools for integrated screening and assessment for mental health conditions; trainings of HCPs; workflows and pathways that integrate mental health interventions within other NCD care.

These materials were designed by ICMR-MINDS group with inputs from key stakeholders to ensure relevance and usability. Standard training manuals and field guides and implementation protocols were created to standardize processes and ensure fidelity to the implementation model. Content was tailored to align with the skill level, roles, and responsibilities of each cadre of HCP and the literacy levels of patients/service users. Adoption, adaptation and translation of the material was carried out for Faridabad district.

The adaptation referred to the modification of intervention components and implementation strategies so that they could be delivered within existing health system structures without disrupting routine services. Adaptation did not involve changing the core content of the interventions. Task-shifting was adapted based on cadre roles and existing responsibilities. ASHA workers were assigned roles in community-level identification rather than formal diagnosis. CHOs conducted initial screening using brief tools during routine NCD visits and offered counselling and brief interventions. MOs performed clinical assessment, initiated treatment, and managed referrals. This adaptation ensured that tasks matched skill levels of each cadre.

Referral pathways were adapted to reduce patient travel and system overload. Instead of routine referral of all screen-positive cases to higher centres, a stepped-care approach was adopted. Mild and moderate cases were managed at AAM-SC, AAM-PHC and CHC. Only severe or complex cases were referred to the District Hospital or specialist services. Tele-consultation options such as TeleMANAS were incorporated as an intermediate referral step, allowing timely specialist input without physical referral.

In addition, specific content (job aids) was created for use in the Faridabad district. These were designed to address identified barriers (e.g., lack of knowledge, structural gaps) leverage facilitators (e.g., existing trust in HCPs, digital readiness) and implement ERIC strategies such as conduct ongoing training, developing educational materials, facilitating the relay of clinical data, audit and feedback, modeling and simulating change and tailoring strategies.

Draft materials were developed using plain language principles and were informed by adult learning techniques. Where appropriate, visual aids and role-play scenarios were included to enhance comprehension and engagement during training. Feedback from experts was incorporated into the respective materials including various study forms, training content and facilitator guides; digital platform and smartphone app (ICMR-MINDS CDSS and dashboard), content aimed at patient education and engagement to raise awareness and promote shared decision-making, and instruction, monitoring and supervision mechanisms.

**Figure 3:**
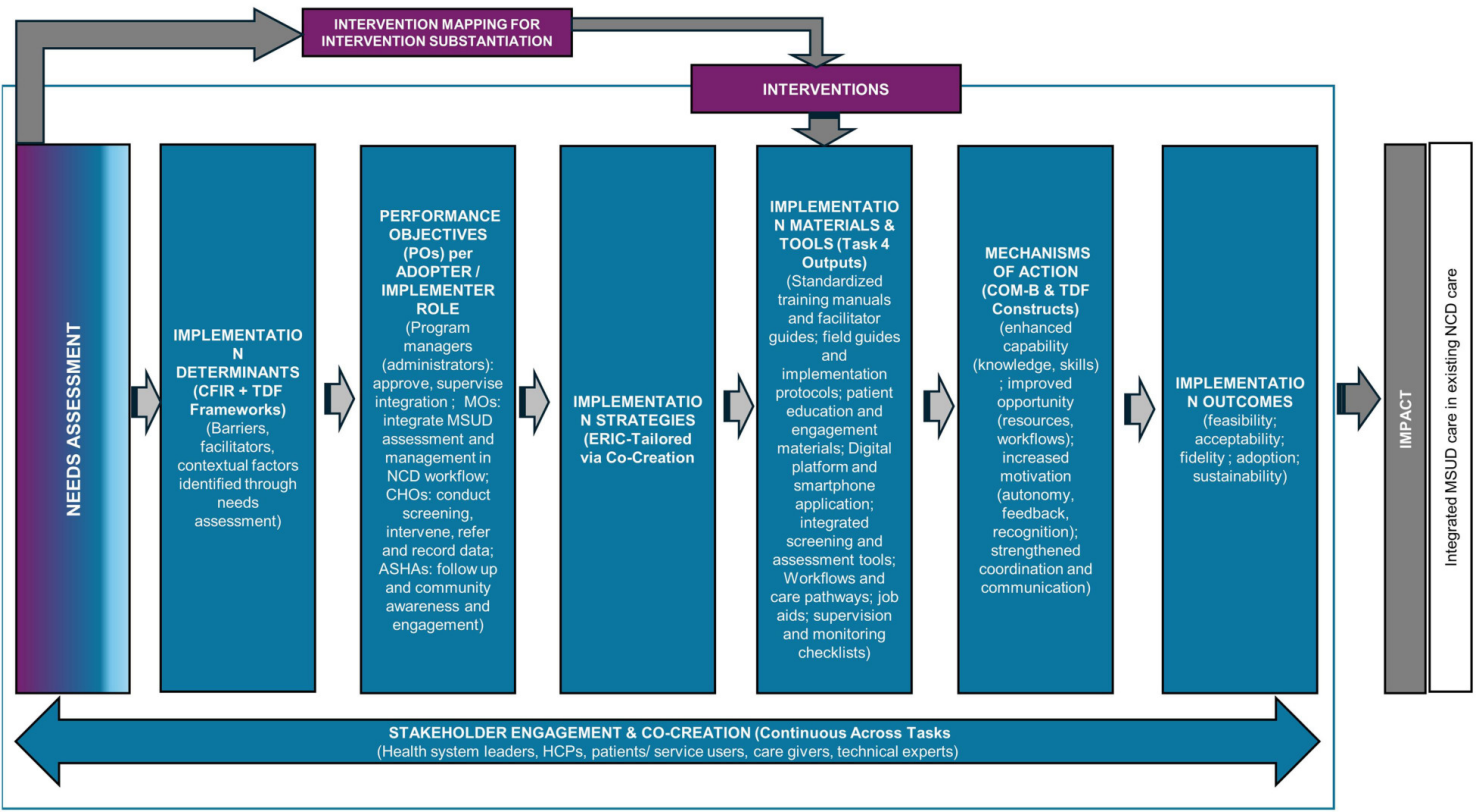
Process of development of implementation strategies for implementation model M0.

Co-creation meetings focused on operationalization and prototype development. Stakeholders reviewed draft tools such as screening and assessment protocols, referral templates, and training modules. They provided feedback on language, usability, and cultural appropriateness. This collaborative design intended to ensure that all implementation materials were feasible for use. All materials were pilot-tested with a small group of end users in Tigaon block for initial feedback. It was incorporated in consultation with technical experts as listed above. Figure 3 presents a graphical depiction of the process of development of implementation strategies for implementation Model M0.

## 4 Discussion

This study presents a comprehensive and systematic approach to the development of implementation strategies aimed at integrating MSUD care into the existing NCD care within the public health system in Faridabad district of Haryana. This contributed to the development of the model M0 for the project. A co-creation approach involving an iterative process was used. In addition, the empirical evidence and contextual realities were at the core of the process.

### 4.1 What this study adds beyond existing integration models

Most existing models for integrating MSUD care into primary care in LMIC focus mainly on clinical effectiveness or describe small-scale pilot programmes (35). These models show that integration is possible. However, they often provide limited guidance on how such integration can be implemented and sustained within routine public health systems (36). The current initiatives include Tele-MANAS (National Tele Mental Health Programme), PRIME (Programme for Improving Mental health carE) and VISHRAM [Vidarbha Stress and Health Programme,(37, 38)]. These are aimed at strengthening MSUD services in India. Besides strengthening the MSUD care, the current work also aimed at MSUD and other NCD care integration. It also operationalised CFIR and IMap for implementing scalable, embedded interventions in public health care.

This study adds to the existing literature by shifting the focus from what should be integrated to how integration can be implemented. The study systematically identified implementation determinants and linked them to theory-informed strategies. Thus, it demonstrated a practical pathway for integrating MSUD care within the existing NCD care. This responds to long-standing recommendations for an integrated approach for MSUD and other NCDs (8).

### 4.2 Advancing implementation science in LMIC contexts

This study advances implementation science by demonstrating the practical application of the CFIR and IMap at scale within a public health system in a LMIC.The prior studies in LMICs have documented barriers such as workforce shortages, stigma, limited specialist availability, and weak referral systems (37, 39). However, most of these studies have stopped at problem identification without translating findings into structured implementation strategies. By using mixed-methods findings to co-create strategies this study bridges the gap between evidence and practice. This aligns with recent calls for context-sensitive, theory-driven implementation research that explicitly addresses system constraints common in LMICs (18, 40).

The integration of stakeholder perspectives was crucial for contextualizing implementation strategies. This ensured that strategies were tailored to the capacity, roles, and expectations of different health system actors. The use of Mendelow's Matrix and IAP2 guidelines ensured that a systematic and participatory approach to co-creation was followed (30, 31). The resulting strategies were designed to address barriers and leverage facilitators. Also, these intended to enhance the acceptability, appropriateness, feasibility, and sustainability of the implementation model.

The use of the step- wise process, together with an iterative method allowed for continuous learning and adaptation. This is of paramount importance in a complex system like India's public health infrastructure. The variation across facilities required tailored approaches.

Finally, the implementation materials were produced with consideration for health care professionals' roles, literacy levels, and local language preferences. This approach aligns with best practices in adult learning and health communication. This is expected to contribute to the accessibility and usability of the materials (41).

The successful adoption of this implementation model in a district with known infrastructural gaps would support its potential for replication. Future scale-up could benefit from existing National Health Mission (NHM) infrastructure and digital health initiatives such as Ayushman Bharat Digital Mission (ABDM). However, this would be subject to the findings of the evaluation of the project.

### 4.3 Alignment with and divergence from prior literature

Our findings confirm well-documented barriers to integrating MSUD care into primary care. Previous research had documented low provider confidence, stigma, lack of private space, weak referral systems, and limited availability of essential medicines. Like previous initiatives such as PRIME and VISHRAM, this study also found that primary care platforms offer strong opportunities for integration when supported by appropriate system-level changes (37, 38).

However, this study diverges from much of the existing literature by moving beyond descriptive findings to explicit development of implementation strategies. This is unlike many previous studies that relied on externally designed or top-down models. The strategies in this study were co-created with stakeholders across system levels. This approach reflects emerging evidence that co-design improves acceptability, feasibility, and sustainability (42).

### 4.4 Policy implications

These findings have potential implications for health policy and programmatic planning. This is particularly true in the context of India and other LMICs facing similar health system challenges. The findings offer actionable insights for policymakers aiming to strengthen the NCD care in the public health system by integrating the MSUD care. Some of the strategies developed as part of this work (e.g., obtaining formal commitments, organizing clinician implementation team meetings, developing academic partnerships, building a coalition) were targeted at policy makers and other health system leaders (state and district health authorities). It also offers a blueprint for converging vertical national programs on NCDs and MSUD.

The structured, stakeholder-informed development of implementation strategies offers a scalable and replicable pathway for integrating MSUD services into existing NCD care. This aligns with national goals under India's Ayushman Bharat program and the National Mental Health Programme (NMHP). The model developed through this study is grounded in empirical data, co-creation, and implementation science frameworks. It can serve as a blueprint for similar such integration.

The study also demonstrated that implementation science (using ERIC, CFIR, and IMap) can be successfully embedded into public health planning. This supports a shift in focus from *ad hoc* program rollouts to evidence-informed policy design. This shall contribute towards ensuring that interventions are not only effective in theory, but also implementable in real-world settings.

Finally, the study demonstrated the feasibility of creation of implementation strategies for Model M0 in a weaker performing district. In addition, the district had a mixed rural- urban demographics. If proven effective, this can potentially contribute to state-level scale-up and add value to national and international discourse on integrating MSUD care into Universal Health Coverage (UHC) goals. This shall help move closer to achieving the Target 3.4 under the Sustainable Development Goal (SDG 3) of reducing premature mortality from NCDs by one-third by 2030 through prevention and treatment, while also promoting mental health and well-being.

### 4.5 Limitations

There are certain limitations of the current article. First, the strategies are targeted and context-specific. This was critical to ensure their appropriateness, feasibility, and acceptability. The article has reported on the creation of implementation strategies as part of Model 0. It did not intend to report on the effectiveness or outcomes of these implementation strategies or the Model M0. Future work under the ICMR-MINDS project in the district will address the full implementation and evaluation phase.

The stakeholder engagement was robust. However, the absence of FGDs with patients/service users and MOs due to logistical constraints may have limited the depth of the input. Although, IDIs were conducted for patients/service users and MOs.

Also, there are certain contextual limitations of this study including challenges posed by the systemic fragmentation of the health system; resource constraints such as limited infrastructure, workforce shortages, medication stock-outs, and digital literacy disparities; policy and governance constraints; stigma and community engagement related challenges. We anticipate amendments and refinements to the Model M0 model as the study progresses.

Despite these limitations, the study makes a significant contribution to implementation science in India and other LMICs. Despite the call to increase focus on implementation research there has been limited response to the same, especially in LMIC. Moreover, there are no reported findings for development of implementation strategies for integrating MSUD care into the existing NCD care. This article demonstrates how an implementation science-informed approach can be a step towards complex service integration efforts. This study directly addresses the global mandate to integrate mental health into primary care settings as outlined in WHO's Mental Health Action Plan 2013–2030. The operationalization of ERIC strategies in Faridabad district aligns with the Lancet Commission's call for context-responsive, system-integrated mental health models in LMICs (45). While pilots have been trialed in countries like Kenya, Nigeria, and Nepal, few have taken a comprehensive, theory-driven approach to implementation strategy development (43–46). This study contributes to the global literature by linking stakeholder-guided ERIC strategies to system-level determinants and roles. This work can serve as a model for other LMICs seeking to integrate MSUD care into the existing NCD care framework into health systems.

## 5 Conclusions

This study addresses a critical gap in India's public health system in the context of integrated care for MSUDs within other NCD programs. The study developed 51 tailored implementation strategies using a co-creation approach and implementation science frameworks to integrate MSUD services into existing NCD care in Faridabad district. Each strategy was linked with specific actions and assigned stakeholders and each of the strategies were mapped to the five innovations (interventions) for the project. This led to the development of implementation model M0 for the district. The approach emphasizes participatory design, and theory-informed planning to address systemic challenges and leverage on the facilitators. This provides a potential framework for resource-constrained settings. This also aligns with national health goals to improve integrated care delivery. Further adoption and real-world evaluation are needed for sustained scale-up and effectiveness. This would be carried out in the next stages of the project.

## Data Availability

The original contributions presented in the study are included in the article/Supplementary Material, further inquiries can be directed to the corresponding author.
