# Supplementary material for: Integrating screening and management of mental disorders, including substance use disorders into other non-communicable disease care: insights from theory-informed implementation strategies creation for implementation model M0 in Faridabad, India as part of ICMR-MINDS project
Source: Front Health Serv. 2026 Apr 23;6:1764829. doi: 10.3389/frhs.2026.1764829 (PMC13149369; doi:10.3389/frhs.2026.1764829)
Supplement: Supplementary file 1 [file Datasheet1.pdf]

**Supplementary Table S1. Examples of the co-creation meetings held with various stakeholders from health system in Faridabad district of Haryana state**	
**Participants**	**Venue**
Deputy District Civil Surgeon cum Nodal Officer for National Mental Health Program, Faridabad District	District Hospital, Faridabad
Civil Surgeon (Chief Medical Officer), Faridabad District Deputy District Civil Surgeon cum Nodal Officer for National Mental Health Program, Faridabad District	District Hospital, Faridabad
Senior Medical Officer, CHC Tigaon	CHC, Tigaon
Medical Officers	CHC, Tigaon
Community Health Officers (CHO), Accredited Social Health Activists (ASHA)	AAM-SC
Chief Secretary of Haryana, Additional Chief Secretary Health Haryana, Director NHM, State Program Officers (NCD, MH) & other key stakeholders of state, DG ICMR, Division Head & Program officer, ICMR, ICMR technical team	Secretariat, Panchkula, Haryana
DGHS Haryana, Civil Surgeon Faridabad, State program officer, NCD,, ICMR technical Team	Secretariat, Panchkula, Haryana
CHO- Community Health Officers (CHO); ASHA- Accredited Social Health Activists; DGHS- Director General of Health Services; NCD- Non Communicable Diseases; MH- Mental Health; AAM PHC- Aayushman Aaarogya Mandir Primary Health Centre; CHC- Community Health Centre; ICMR- Indian Council of Medical Research; DG- Director General	

**Supplementary Table S2. Implementation factor and corresponding CFIR domain and Determinant (arranged alphabetically)**		
**Implementation factor**	**CFIR domain**	**Determinant**
Access to knowledge and information	Inner setting	Information flow<br>Training adequacy
Adaptability	Innovations	Innovation compatibility<br>Self-efficacy
Available resources	Inner setting	Environmental conditions (structural, organizational) such as resource availability, organizational capacity
Community engagement	Outer setting	Collective efficacy<br>Social norms<br>Trust
Complexity	Innovations	Cognitive load<br>Perceived behavioral control
Continuity of care (referral pathways)	Inner setting	Environmental conditions (structural, organizational)
Cost	Innovations	Perceived value<br>Resource constraints
Culture	Inner setting	Norms<br>Organizational values
Design quality and packaging	Innovations	Aesthetic appeal<br>Comprehension<br>Usability
Evidence strength and quality	Innovations	Beliefs<br>Attitudes<br>Perceived usefulness
End beneficiaries	Innovations	Innovation's compatibility
Engagement of stakeholders	Process	Attitude<br>Beliefs<br>Motivation
Executing	Process	Feasibility<br>Fidelity
External policies and incentives	Outer setting	Policy environment<br>External motivation
Feedback, Evaluation and monitoring	Process	Feedback and reinforcement<br>Monitoring
Implementation climate	Inner setting	Change valence<br>Organizational readiness
Knowledge, self efficacy, skill set, personal attributes and stage of change of the health care professionals	Individuals	Knowledge<br>Skills<br>Attitudes<br>Beliefs<br>Motivation<br>Transtheoretical model constructs
Leadership engagement (administrative authorities)	Inner setting	Commitment<br>Organizational commitment<br>Leadership
Patient needs and resources	Outer setting	Health beliefs<br>Cultural norms<br>Accessibility
Planning	Process	Goal clarity<br>Strategic alignment

Prioritisation	Inner setting/ outer setting	Attitudes<br>Beliefs<br>Organizational commitment
Structural characteristics	Inner setting	Environmental conditions<br>Infrastructure

Supplementary Table S3. Cross-walk (matrix) of outcomes, adopters/implementers, POs, and determinants

Outcome	Adopter / Implementer	Performance Objective (PO)	Determinants (CFIR / TDF domains)
Adoption outcome- Integration of MSUD services into existing NCD care	District Health managers (administrators)	Approves and endorses integrated service delivery within district NCD program plans.	*Outer setting:* leadership engagement, policy alignment; *Process:* planning, external collaboration.
	Medical Officer (MO)	Incorporates MSUD screening tools into routine NCD consultations and supervises their use by CHOs.	*Inner setting:* compatibility, resource availability; *TDF:* knowledge, skills, professional role, beliefs about capabilities.
Implementation outcome- Fidelity to screening, referral, and follow-up workflows	Community Health Officer (CHO)	Conducts standardized MSUD screening for all NCD patients during visits.	*TDF:* knowledge, skills, self-efficacy; *CFIR:* access to training, relative advantage, workflow fit.
	Accredited Social Health Activist (ASHA)	Provides follow-up and adherence support to patients referred for MSUD management.	*CFIR:* communication networks, peer support; *TDF:* social influence, reinforcement.
Implementation outcome- Acceptability and feasibility of the integrated model	Community Health Officer (CHO) / MO	Documents MSUD-related data in the digital platform.	*Inner setting:* data system readiness, available resources; *TDF:* memory, attention, and decision processes.

Implementation outcome- Sustainability of integrated MSUD–NCD care	Program managers (administrators)	Reviews monthly data and provides feedback to facilities on screening, management coverage and referral compliance.	*Process:* reflecting and evaluating, feedback mechanisms; *TDF:*reinforcement, behavioral regulation.
	ASAH, CHO, MO	Participate in periodic refresher training and peer-support sessions.	*TDF:* motivation, professional identity, behavioral regulation; *CFIR:*learning climate, leadership support.
The list provided here is not all inclusive and offers some examples for the purpose of demonstration of the cross-walk (matrices).<br>MSUD- Mental disorders, including substance use disorders; DH- District Hospital; CHO- Community Health Officers (CHO); ASHA- Accredited Social Health Activists; DGHS- Director General of Health Services; NCD-Non Communicable Diseases; MH- Mental Health; AAM PHC- Aayushman Aaarogya Mandir Primary Health Centre; CHC- Community Health Centre; ICMR- Indian Council of Medical Research; DG- Director General; HCP- Health Care professionals; MO- Medical Officers; CFIR- Consolidated Framework For Implementation Research; TDF- Theoretical Domains Framework			

Supplementary Table S4. Performance objectives, Determinant and Change Objectives (arranged in alphabetical order for Determinant		
**Performance Objective**	**Determinant**	**Change Objectives**
Establish collaborations with academic institutions	**Academic collaboration**	Strengthen capacity by involving academic institutions for training, mentoring, and evaluation support
Improve service access for clients	**Accessibility**	Reduce physical, linguistic, and financial barriers to access
Enhance usability and appeal of materials	**Aesthetic appeal**	Use visually engaging and well-organized design for intervention tools
Ensure sufficient staff, tools, and space	**Available resources**	Mobilize and allocate adequate physical, financial, and human resources
Address stakeholder attitudes	**Attitudes**	Engage in two-way communication to address doubts and misconceptions
Foster belief in evidence strength	**Beliefs**	Present robust evidence from credible sources and local pilots
Increase community involvement	**Collective efficacy**	Build local networks and shared goals to drive participation and support
Improve comprehension of tools/protocols	**Comprehension**	Use plain language, visuals, and repetition to increase understanding
Strengthen referral systems	**Continuity of care (referral pathways)**	Strengthen partnerships and streamline patient transfer procedures
Reduce complexity of new procedures	**Cognitive load**	Simplify and streamline workflows to avoid overwhelming staff
Generate readiness and buy-in	**Change valence**	Communicate the need for change and its alignment with shared goals
Cue health worker actions through prompts	**Cueing Behavioral triggers**	Support timely clinical decision-making through point-of-care reminders
Strengthen underlying infrastructure	**Environmental conditions Infrastructure**	Upgrade physical and technical infrastructure necessary for implementation
Optimize clinical workflow efficiency	**Environmental conditions (workflow/process)**	Reduce bottlenecks and improve flow by streamlining clinical pathways

Incentivize performance and uptake	**External motivation**	Leverage policy mandates or financial incentives to boost engagement
Establish systems for learning and feedback	**Feedback and reinforcement**	Set up continuous, actionable feedback loops
Support practical execution efforts	**Feasibility**	Provide clear, step-by-step guidelines and resources for implementation
Maintain adherence to the original design	**Fidelity**	Monitor implementation regularly and correct deviations from protocol
Align planning with organizational goals	**Goal clarity**	Define measurable and clearly communicated goals
Address client needs and beliefs	**Health beliefs**	Ensure services reflect patient values, perceptions, and expectations
Improve access to and sharing of information	**Information flow**	Strengthen internal communication channels and training delivery mechanisms
Build belief in intervention alignment	**Innovation compatibility**	Show how the intervention supports staff roles, values, and local practice
Ensure intervention fits end users' needs	**Innovation's compatibility**	Co-design features that match end users' preferences and daily routines
Develop individual competencies	**Knowledge, Skills, Attitudes, Beliefs, Motivation**	Offer training aligned with the Transtheoretical Model stages of change
Strengthen leadership capacity	**Leadership**	Train and support managers (administrators) to champion and facilitate implementation
Enable effective tracking of progress	**Monitoring**	Use simple and efficient monitoring tools and dashboards
Motivate stakeholder participation	**Motivation**	Clarify personal and organizational benefits of involvement
Align intervention with organizational culture	**Norms**	Reinforce shared organizational norms that support the intervention
Promote alignment with core values	**Organizational values**	Communicate how the intervention supports organizational missions and ethics
Secure leadership involvement	**Organizational commitment**	Obtain visible endorsement and active involvement from leadership

Justify resource investments	**Perceived value**	Demonstrate cost-benefit rationale to stakeholders and leadership
Improve control over task performance	**Perceived behavioral control**	Empower staff with tools, autonomy, and clear roles for implementation tasks
Promote perceived usefulness of intervention	**Perceived usefulness**	Share success stories and testimonials from similar settings
Promote perceived usefulness of intervention	**Perceived usefulness Health beliefs**	Share success stories, testimonials, and demonstrate alignment with user health values
Align with external policy landscape	**Policy environment**	Map intervention activities to relevant national and local policies
Promote prioritization of the intervention	**Prioritisation**	Ensure leaders and staff allocate time and resources to support the program
Minimize implementation barriers	**Resource constraints**	Identify and address specific material or financial gaps
Integrate into organizational strategy	**Strategic alignment**	Embed the intervention within the broader institutional strategy
Build efficient referral systems	**Structural/organizational conditions**	Develop structured pathways and clear protocols for client referrals
Support stage-based staff development	**Transtheoretical model constructs**	Tailor support based on where individuals are in their behavior change journey
Ensure staff are adequately trained	**Training adequacy**	Provide continuous, context-relevant training sessions for health care professionals
Strengthen community-provider trust	**Trust**	Build relationships and transparency through sustained engagement efforts
Design for ease of use	**Usability**	Involve users in testing and refining tools for intuitive use

Supplementary Table S5. Determinant, Relevant theoretical method and Implementation strategy (Based on Expert Recommendations for Implementing Change (ERIC))

Determinant	Relevant Theoretical Method	Implementation Strategy (ERIC Taxonomy)
Academic collaboration	Academic partnership theory	Develop academic partnerships<br><br>Provide ongoing consultation<br><br>Work with educational institutions<br><br>Work with educational institutions
Information flow	Knowledge management<br>Learning theories	Conduct educational meetings<br><br>Organize clinician implementation team meetings<br><br>Create a learning collaborative
Training adequacy	Adult learning theory<br>Social Cognitive Theory	Conduct ongoing training<br><br>Distribute educational materials<br><br>Make training dynamic<br><br>Organize clinician implementation team meetings
Innovation compatibility	Diffusion of Innovations Theory	Promote adaptability<br><br>Provide clinical supervision<br><br>Tailor strategies
Self-efficacy	Self-regulation theory<br>Social modeling	Identify and prepare champions<br><br>Model and simulate change
Resource availability<br>Organizational capacity	Organizational change theory	Conduct local needs assessment<br><br>Create new clinical teams<br><br>Develop academic partnerships<br><br>Develop resource sharing agreements<br><br>Facilitate relay of clinical data to providers<br><br>Revise professional roles<br><br>Use data warehousing techniques
Cueing behavioral triggers	Cueing theory<br>Cognitive support tools	Remind clinicians<br><br>Organize clinician implementation team meetings

Collective efficacy	Social network theory	Build a coalition<br><br>Organize clinician implementation team meetings<br><br>Promote network weaving<br><br>Create a learning collaborative
Social norms	Normative re-education theory	Organize clinician implementation team meetings<br><br>Use opinion leaders
Trust	Relational contracting	Use advisory boards/workgroups
Cognitive load	Cognitive load theory	Change record systems<br><br>Facilitation<br><br>Model and simulate change<br><br>Provide clinical supervision<br><br>Remind clinicians<br><br>Tailor strategies
Perceived behavioral control	Theory of Planned Behavior	Tailor strategies
Structural/organizational conditions	Theories of Social Networks and Social Support	Change physical structure and equipment
Perceived value	Health economics<br><br>Value framing	Access new funding
Resource constraints	Resource optimization theory	Use other payment schemes
Norms	Organizational culture theory	Use opinion leaders
Organizational values	Organizational identity theory	Identify and prepare champions<br><br>Model and simulate change

Aesthetic appeal	Persuasive communication theory	Make training dynamic<br><br>Promote adaptability<br><br>Tailor strategies
Comprehension	Plain language and elaboration theory	Develop educational materials
Usability	User-centered design theory	Purposely re-examine the implementation
Beliefs	Theory of Reasoned Action	Distribute educational materials<br><br>Provide local technical assistance
Attitudes	Communication-Persuasion Matrix	Intervene with patients/consumers to enhance uptake and adherence<br><br>Obtain and use patients/consumers and family feedback<br><br>Organize clinician implementation team meetings<br><br>Use advisory boards and workgroups<br><br>Use mass media
Motivation	Expectancy-value theory	Identify and prepare champions<br><br>Model and simulate change<br><br>Organize clinician implementation team meetings
Feasibility	Process mapping PDSA cycles	Develop academic partnerships<br><br>Stage implementation scale- up<br><br>Work with educational institutions
Fidelity	Fidelity monitoring theory	Develop academic partnerships<br><br>Audit and provide feedback<br><br>Provide ongoing consultation
Policy environment	Advocacy theory Regulatory alignment	Obtain formal commitments
External motivation	Incentivization theory	Alter incentive/allowance structures

Feedback and reinforcement	Social Cognitive Theory Goal-setting theory	Develop and implement tools for quality monitoring<br><br>Develop and organize quality monitoring systems<br><br>Obtain and use patients/consumers and family feedback<br><br>Organize clinician implementation team meetings<br><br>Provide ongoing consultation
Monitoring	Continuous quality improvement	Develop and implement tools for quality monitoring<br><br>Develop and organize quality monitoring systems<br><br>Obtain and use patients/consumers and family feedback<br><br>Create a learning collaborative<br><br>Organize clinician implementation team meetings
Change valence	Value clarification theory	Conduct local consensus discussions<br><br>Obtain formal commitments
Organizational readiness	Organizational readiness theory	Assess for readiness and identify barriers and facilitators<br><br>Develop a formal implementation blueprint
Knowledge, skills, attitudes, beliefs, motivation	Transtheoretical Model Theories of learning	Facilitation<br><br>Provide ongoing consultation<br><br>Shadow other experts<br><br>Visit other sites
Leadership	Transformational leadership theory	Inform local opinion leaders<br><br>Obtain formal commitments
Perceived usefulness<br>Health beliefs	Health communication and behavior change theories	Intervene with patients/consumers to enhance uptake and adherence<br><br>Obtain and use patients/consumers and family feedback<br><br>Prepare patients/consumers to be active participants
Health beliefs	Health Belief Model	Intervene with patients/consumers to enhance uptake and adherence<br><br>Obtain and use patients/consumers and family feedback<br><br>Prepare patients/consumers to be active participants<br><br>Tailor strategies

Cultural norms	Cultural tailoring theory	Capture and share local knowledge<br><br>Obtain and use patients/consumers and family feedback<br><br>Prepare patients/consumers to be active participants
Accessibility	Barrier reduction theory	Change physical structure and equipment<br><br>Obtain and use patients/consumers and family feedback<br><br>Intervene with patients/consumers to enhance uptake and adherence
Goal clarity	Goal-setting theory	Develop a formal implementation blueprint
Strategic alignment	Organizational alignment theory	Develop a formal implementation blueprint
Attitudes<br>Beliefs<br>Organizational commitment	Organizational Development Theory	Tailor strategies<br><br>Inform local opinion leaders<br><br>Obtain formal commitments
Environmental conditions Infrastructure	Infrastructure assessment and improvement theory	Change physical structure and equipment
Environmental conditions (workflow/process)	Process reengineering, Lean methods	Assess for readiness and identify barriers and facilitators<br><br>Conduct cyclical small tests of change<br><br>Change physical structure and equipment<br><br>Change record systems<br><br>Facilitation

Supplementary Table S6. Implementation strategy (Based on Expert Recommendations for Implementing Change (ERIC) taxonomy), Specific actions and target actors (stakeholders)		
**Implementation strategy (Based on Expert Recommendations for Implementing Change (ERIC))**	**Specific actions**	**Target actors (stakeholders)**
**Access new funding**	Secure ongoing grant from funding agency for the project<br><br>Ensure budget allocation to make medicine to treat MSUD available at public health facilities	AIIMS New Delhi project team, Health system leaders (policy makers and state and district health authorities)
**Assess for readiness and identify barriers and facilitators**	Assess and analyse current level of integration<br><br>Redefine organizational ethos/culture to support innovations and new interventions	Health system leaders (policy makers and state and district health authorities)
**Audit and provide feedback**	Review implementation fidelity and outcomes and share data with teams	Health system leaders (policy makers and state and district health authorities), HCPs
**Build a coalition**	Organize multisectoral alliances to promote and oversee implementation	Health system leaders (policy makers and state and district health authorities), HCPs
**Capture and share local knowledge**	Capture local knowledge from HCPs at the public health facilities on how they made something work in their setting and then share it with other sites	Health system leaders (policy makers and state and district health authorities), HCPs
**Provide local technical assistance**	Develop and use a system located at AIIMS New Delhi and ICMR HQ to deliver technical assistance focused on implementation issues<br><br>Provide hand holding and troubleshooting with using the innovations (interventions)<br><br>Offer on- site technical support and trouble shooting for the digital digital platform and smartphone app	ICMR technical team; AIIMS New Delhi Project Team
**Change physical structure and equipment**	Update facility layout to support new workflows	Health system leaders (policy makers and state and district health authorities), HCPs
**Change record systems**	Create system integrated into the digital platform and smartphone app that can be subsequently integrated into the state HMIS	Health system leaders (state and district health authorities), Digital platform and smartphone app (ICMR-MINDS CDSS and dashboard) development team
**Conduct cyclical small tests of change**	Pilot tools, gather feedback, revise, and repeat	HCPs, ICMR technical Team

**Conduct educational meetings**	Organize sessions to introduce innovations (interventions) and share implementation plans	Health system leaders (policy makers and state and district health authorities), HCPs
**Conduct local consensus discussions**	Conduct discussions with Health system leaders (policy makers and state and district health authorities), HCPs to agree on core problems, needs, and goals,<br>and to assess the benefit of implementing the innovations (interventions)	Health system leaders (policy makers and state and district health authorities), HCPs
**Conduct local needs assessment**	Use surveys and interviews to assess population needs and system readiness	Health system leaders (policy makers and state and district health authorities), HCPs, Patients/service users
**Conduct ongoing training**	Deliver induction, refresher, and follow-up sessions on using innovations (interventions)	HCPs
**Create a learning collaborative**	Establish a multi-facility learning collaborative, share lessons learned across health facilities, use collective feedback to adapt implementation strategies	HCPs
**Create new clinical teams**	Form multi-disciplinary teams aligned with the intervention goals	Health system leaders (policy makers and state and district health authorities)
**Develop a formal implementation blueprint**	Develop a formal implementation blueprint including aim/purpose of the implementation; scope of the change; timeframe and milestones; appropriate performance/progress measures.	Health system leaders (policy makers and state and district health authorities)
**Develop academic partnerships**	Collaborate with academic institutions for training, research, and evaluation support	Health system leaders (policy makers and state and district health authorities)
**Develop and implement tools for quality monitoring**	Develop tools for quality monitoring<br><br>Continuously assess the progress of the implementation and adjust when indicated	ICMR technical Team, AIIMS New Delhi project team
**Develop and organize quality monitoring systems**	Use digital dashboard created as part of the project to monitor quality	ICMR technical Team, AIIMS New Delhi project team
**Develop educational materials**	Simplify language, layout, and structure of materials for users	AIIMS New Delhi project team, ICMR-MINDS
**Develop resource sharing agreements**	Set up formal agreements between facilities to share personnel or tools	Health system leaders (policy makers and state and district health authorities), ICMR technical Team
**Distribute educational materials**	Develop and share guides, presentations, and other learning resources	HCPs

**Facilitate relay of clinical data to providers**	Develop tools for smooth transfer of clinical info across facilities	Digital platform and smartphone app development team (ICMR-MINDS CDSS and dashboard)
**Facilitation**	Provide hand holding and troubleshooting with using the innovations (interventions) to support adoption	HCPs
**Identify and prepare champions**	Select motivated staff to lead and encourage adoption	HCPs
**Inform local opinion leaders**	Organize meetings with health system leaders to introduce the innovations<br><br>Perform open discussions with health system leaders to discuss objections, concerns, change their attitude/ perspective, and guide them toward adopting the intervention	Health system leaders (policy makers and state and district health authorities)
**Intervene with patients/consumers to enhance uptake and adherence**	Include patients/ service users and care givers in planning, decision-making, and implementation	Patients/ service users and care givers
**Make training dynamic**	Use interactive, participatory methods to increase engagement and retention	HCPs
**Model and simulate change**	Conduct mock trials, role plays, or simulations to prepare staff	HCPs
**Obtain and use patients/consumers and family feedback**	Make patient/ service users and care givers part of the advisory boards and workgroups	Patients/ service users, care givers
**Obtain formal commitments**	Obtain formal written commitments from o health system leaders to implement the model<br><br>Make a public declaration/announcement expressing organizational leaders' determination to implement the model	Health system leaders (policy makers and state and district health authorities)
**Organize clinician implementation team meetings**	Periodically organised meetings of the HCPs and other stakeholders	HCPs
**Prepare patients/consumers to be active participants**	Assess the needs of the target population in Faridabad district<br><br>Activate stakeholder groups<br><br>Focus on promoting participation as part of the awareness sessions	Patient/ service users and care givers
**Promote adaptability**	Modify intervention components to fit local capacity and resources	Health system leaders (policy makers and state and district health authorities)

**Provide clinical supervision**	Conduct regular mentoring and check-ins with frontline staff	Health system leaders (policy makers and state and district health authorities)
**Provide ongoing consultation**	Provide ongoing consultation with experts in the strategies used to support implementing the innovation	Health system leaders (policy makers and state and district health authorities), HCPs
**Promote network weaving**	Encourage peer support, knowledge exchange, and cross-facility learning	HCPs
**Purposely re-examine the implementation**	Continuously refine materials based on real-world use	Digital platform and smartphone app development team (ICMR-MINDS CDSS and dashboard), AIIMS New Delhi Project team
**Remind clinicians**	Use point-of-care prompts like posters, checklists, or app notifications	HCPs
**Revise professional roles**	Reassign tasks and create new role definitions to support implementation	Health system leaders (policy makers and state and district health authorities)
**Shadow other experts**	Arrange observation sessions at facilities where intervention is implemented to obtain direct observation<br><br>Organise meetings with the HCPs form the facilities where the intervention is implemented	HCPs
**Stage implementation scale up**	Phase implementation efforts by starting in one block and then extending to a system wide rollout	AIIMS New Delhi Project Team
**Tailor strategies**	Adapt strategies based on local context, needs, and feedback	HCPs
**Use advisory boards and workgroups**	Identify relevant stakeholders<br><br>Map stakeholders by their influence, power, authority, and importance in relation to implementation using the Mendelow's matrix<br><br>Activate stakeholder groups	All stakeholders
**Use data experts**	Involve experts to inform management on the use of data generated by implementation efforts	ICMR technical team
**Use data warehousing techniques**	Aggregate and organize health data for access and analysis	Digital platform and smartphone app development team (ICMR-MINDS CDSS and dashboard)
**Use mass media**	Spread information about the implementation using IEC material	Health system leaders (policy makers and state and district health authorities), HCPs
**Use opinion leaders**	Identify respected individuals to influence peer adoption	Health system leaders (policy makers and state and district health authorities), HCPs
**Use other payment schemes**	Do not transfer the cost of intervention to the HCPs and patients/ service users	ICMR, AIIMS New Delhi Project team

**Visit other sites**	Arrange study visits to facilities where intervention is implemented<br>to obtain direct observation<br><br>Organise meetings with the HCPs form the facilities where the intervention is implemented	HCPs
**Work with educational institutions**	Establish collaboration with educational institutions to train HCPs in the innovation	AIIMS New Delhi Project team
Health system leaders refer to persons involved in policy, planning, budget allocation and administration of the health system in the state of Haryana and Faridabad district.<br><br>The AIIMS New Delhi project team was involved in all the actions. The name of the team has been specifically mentioned against some of the actions where the other actors (stakeholders) were not primarily involved in that action.<br><br>MSUD- Mental disorders, including substance use disorders; DH- District Hospital; CHO- Community Health Officers (CHO); ASHA- Accredited Social Health Activists; DGHS- Director General of Health Services; NCD- Non Communicable Diseases; MH- Mental Health; AAM PHC- Aayushman Aaarogya Mandir Primary Health Centre; CHC- Community Health Centre; ICMR- Indian Council of Medical Research; DG- Director General; HCP- Health Care professionals; MO- Medical Officers; CFIR- Consolidated Framework For Implementation Research; TDF- Theoretical Domains Framework		
